# Up to You and up to Them—Achievement and Power Motives as Building Blocks of Leadership Potential and Overall Reputation

**DOI:** 10.3390/bs16010131

**Published:** 2026-01-16

**Authors:** Antun Palanović, Nataša Trojak, Zvonimir Galić

**Affiliations:** 1 Academic Unit for Psychology, Algebra Bernays University, 10000 Zagreb, Croatia; natasa.trojak@algebra.hr; 2 Department of Psychology, University of Zagreb, 10000 Zagreb, Croatia; zgalic@ffzg.hr

**Keywords:** achievement motive, power motive, reputation, leadership, signalling theory, role congruity theory

## Abstract

Background: Building on socioanalytic theory and signaling theory, this study examined whether self-reported motives—representing individuals’ identities—translate into reputational outcomes. Furthermore, drawing on role congruity theory, the study examined whether gender would moderate the strength of the relationship between the self-reported motives and reputational consequences. Methods: We used a large sample of management students (*N* = 349) on which we collected self-reported achievement and power motives, and peer ratings (*N* = 508) of overall reputation and leadership potential. Results: We found that (a) achievement motive was positively associated with leadership potential and overall reputation (including trustworthiness and competence); (b) power motive was positively associated with leadership potential; and (c) both motives were equally strong predictors of leadership potential, but only achievement motive was a significant predictor of overall reputation. Finally, in line with role congruity theory, we observed that the positive associations between both motives and leadership potential were stronger for male than for female students. However, for overall reputation, this applied only to the power motive, suggesting that gender affects how motivational signals are socially interpreted. Conclusions: These findings offer implications for future research and provide practical insights into talent identification, leadership development, and performance evaluation processes.

## 1. Introduction

*“That’s the funny thing about reputation: it’s not up to you. It’s up to them.”*

—Harvey Specter (Suits)

The quote from the television series Suits gets one thing right about reputation—it is “up to them”. Reputation is fundamentally social—shaped by how others perceive an individual’s traits, behavior, and performance over time (Ferris et al., 2003). For instance, socioanalytic theory (Hogan & Holland 2003) views personality from two perspectives: (1) identity, which reflects how we see ourselves, and (2) reputation, which reflects how others evaluate us based on repeated social interactions. A favorable reputation can lead to important career outcomes, such as more promotions and greater influence and autonomy at work (Zinko et al., 2012). At the same time, socioanalytic theory states that reputation is not beyond our control. At least partially, it emerges from stable psychological characteristics that influence how we behave across situations. Personality traits such as conscientiousness, emotional stability, and extraversion, as well as our motives for achievement, power, and affiliation, largely shape our behavior and, in turn, influence our reputation over time. At the same time, self-ascribed psychological characteristics reflect one’s identity—the image or signal a person wants to convey to others. In that sense, reputation is, in addition to being “up to them,“ also “up to you” as an individual’s behavior signals to others what they can expect from him/her

### 1.1. Signaling Theory

Signaling theory suggests that individuals’ behaviors and accomplishments carry important signals about their ability and underlying personality traits (Spence, 1973). For instance, obtaining a university degree might signal that a job applicant has above-average cognitive ability and possesses trait conscientiousness, which should, in turn, translate into better job performance (Caplan, 2018). In an organizational/career context, this also applies to employees signaling their readiness for promotion, or leaders signaling trustworthiness to followers (Connelly et al., 2011).

Signaling theory emphasizes the role of observable behaviors. For instance, Zinko et al. (2007) distinguish between two types of behaviors that shape reputation: human capital (skills and knowledge) and social effectiveness (skills in reading and managing social interactions). However, these behaviors often originate from stable individual differences. As such, understanding the dispositional antecedents of behavior—such as personality traits and motivational tendencies—offers a deeper perspective on how reputations are formed. Leadership literature empirically supports this view, as meta-analytic data show that leader personality traits are distal predictors of leadership effectiveness through specific leadership styles, such as transformational leadership and initiating structure (DeRue et al., 2011). This highlights the importance of examining how specific personality traits, rather than just observable behaviors, contribute to the formation of reputation.

While research on (personality) antecedents of reputation in organizational contexts is scarce, there are important insights from the literature on leader emergence. Leader emergence can serve as a proxy for employee reputation, as it often reflects others’ perceptions of an individual’s observable behaviors that signal leadership potential. Meta-analytical data show that among the self-reported Big Five personality traits, extraversion is the strongest predictor of leadership emergence, followed by openness and conscientiousness (Judge et al., 2002). In other words, people see energetic, sociable, ambitious, organized, and creative individuals as “leadership material”, which, in turn, helps them obtain leadership positions. Perhaps an even more telling example of how personality traits shape reputation—employees high on trait narcissism engage in behaviors that signal confidence and charisma. In turn, they are more likely to obtain leadership positions, but that does not translate into effective leadership (Judge et al., 2002; Nevicka et al., 2018).

Outside of leadership literature, Zinko et al. (2012) conducted two studies with business student and employee samples and found that participants with higher levels of cognitive ability, self-efficacy, and political skill developed better reputations. Furthermore, Kilduff and Day (1994) found that over five years, individuals high in self-monitoring, a tendency to observe and regulate one’s self-presentation, earned significantly more cross-company promotions or internal promotions than low self-monitors. In summary, personality traits are important “building blocks” for signaling competence and, consequently, for building one’s work reputation.

### 1.2. Achievement and Power Motive

Socioanalytic theory (Hogan & Holland, 2003) explains human behavior through the lens of evolutionary psychology. This approach observes that people live and work in groups that have a clear status hierarchy. In such conditions, their behavior is driven by two fundamental motives—the need to get ahead (gaining status and achievement), and the need to get along (being accepted and developing social ties).

The need to get ahead represents the drive to attain status, influence, and success within social hierarchies. This need aligns with two fundamental motives: the achievement and power motives. Achievement motive reflects a desire to meet or exceed standards of excellence, pursue mastery, and demonstrate competence (McClelland, 1985; Ružojčić et al., 2019). Individuals high in this motive tend to set challenging goals, persevere in the face of obstacles, and focus on delivering results—behaviors that are likely to signal competence and high potential to observers. Power motive, in contrast, involves a desire to exert influence, control resources, and attain status within a social hierarchy (Winter, 1973; Galić et al., 2021). Individuals with strong power motives often seek leadership roles, strive for visibility, and engage in behaviors to gain authority and recognition. For example, a recent meta-analysis by Badura et al. (2020) showed that compelling motivation to lead, which can be used as a proxy of power motive, reliably predicts both leader emergence and leadership effectiveness.

This research examines the relationship between achievement/power motives and two reputational outcomes: leadership potential and overall reputation. Leadership potential reflects beliefs about an individual’s future capacity to assume leadership responsibilities (Silzer & Church, 2009). Overall reputation captures the general perception of a person’s competence and reliability (Ferris et al., 2003). Both achievement and power motives should manifest in behaviors that signal ambition, competence, and influence. As such, they are likely to serve as important predictors of how individuals are perceived in terms of their leadership potential and overall reputation. Based on this, we propose the following hypothesis:

**Hypothesis** **1.** *Individuals’ self-reported achievement and power motives will have statistically significant and positive relationships with other-reported leadership potential and overall reputation.*

While both achievement and power motives reflect the broader drive to “get ahead,” they may shape reputation outcomes in slightly different ways. Challenging goals, diligence, and task mastery drive individuals high in achievement motive. This is likely to contribute more broadly to a positive overall reputation, as these behaviors are generally viewed as reliable indicators of competence and effectiveness. In contrast, individuals high on the power motive desire influence and visibility, which may align better with perceptions of leadership potential, as it signals a willingness to take control in group settings. This aligns with the findings of Judge et al. (2002), who found that extraversion, especially the facets of dominance and sociability, were the strongest personality predictors of leadership emergence.

**Hypothesis** **2.** *Achievement motive and power motive will differ in their relationships with leadership potential and overall reputation. Specifically, achievement motive will be a stronger predictor of overall reputation, while power motive will be a stronger predictor of leadership potential.*

### 1.3. Moderating Role of Gender

Achievement and power motives are the key drivers of behaviors that signal readiness to take on challenging tasks, responsibilities, and leadership positions. Hence, people high in these motives are more likely to build a strong reputation. However, social psychology literature suggests things may not be as straightforward (e.g., Heilman & Okimoto, 2007; Williams & Tiedens, 2016). Specifically, it seems that the social rewards for expressing these motives may depend on an individual’s gender. Role congruity theory of prejudice toward female leaders proposes that leadership traits such as assertiveness, dominance, and ambition are more closely aligned with stereotypical male gender roles than with female roles (Eagly & Karau, 2002). Consequently, this leads to the prediction that men are more likely to be viewed favorably than women when they exhibit behaviors stemming from power or achievement motives.

Empirical findings generally support this prediction. Women who display agentic traits associated with the need to get ahead—such as self-promotion, dominance, or strong career ambition—can experience backlash effects. Experimental research showed that female managers received lower ratings of likability, interpersonal hostility, and boss desirability than male managers (Heilman & Okimoto, 2007). Interestingly, the same study found that these adverse effects are mitigated when participants are clearly presented with the information about the communal attributes of the female managers. In another experiment, both men and women who engaged in self-promotion were perceived as more competent. However, only men were perceived as more hirable, and self-promotion decreased women’s social attractiveness (Rudman, 1998). Finally, a meta-analysis by Williams and Tiedens (2016) showed that while men and women who behave dominantly are rated equally on competence, women get lower ratings on likability and hireability.

Thus, while power-motive behaviors may signal leadership potential in men, they may be misinterpreted or even penalized in women. Similarly, ambition, which reflects a mix of achievement and power motives, has been found to predict career success more strongly for men than for women (Judge & Kammeyer-Mueller, 2012). These patterns reflect a sort of “Catch-22” for women in the workplace: they are expected to be communal and modest, yet are judged against standards that reward assertiveness and visibility (Rudman, 1998).

Taken together, these findings indicate that gender moderates the relationship between motivational traits and how others perceive individuals. That is, the same behavior or motivational expression may lead to positive impressions for men but more mixed or even negative impressions for women.

**Hypothesis** **3.** *Gender will moderate the relationships between achievement and power motives, and other-rated leadership potential and overall reputation. Specifically, the positive relationships will be stronger for men than for women.*

Importantly, reputational constructs remain underexplored in Industrial/Organizational psychology despite their relevance for career advancement, performance appraisals, and leadership selection (Ferris et al., 2003; Zinko et al., 2012). As a proof-of-concept study, the present research aims to offer an initial theoretical and empirical framework for understanding how motivational traits shape reputational outcomes, using a sample of management students. Therefore, the contribution of this research is threefold. First, on the theoretical front, it integrates socioanalytic theory, signaling theory, and role congruity theory to explain how self-ascribed motives relate to peer-rated leadership potential and reputation. The three theoretical approaches link the identity to reputation (socioanalytic theory), provide the mechanism for this link (signaling theory), and introduce gender as an important moderator (role congruity theory). Second, empirically, the research combines self-report measures of motivation with peer evaluations of reputational outcomes. Third, by drawing on a large sample of management students, the research might offer insights that can inform both research and practice of talent identification and leadership development. Even though the student sample limits the generalizability of our results, it provides a theoretically relevant context for testing initial hypotheses before extending the research to applied organizational settings.

## 2. Materials and Methods

### 2.1. Sample and Procedure

We recruited 349 management students (*M_age_* = 21.64, *SD_age_* = 2.24, 37% male, 63% female). They completed an online survey and were instructed to recruit raters to assess their leadership potential and overall reputation in a separate online survey. All in all, 258 participants recruited at least one rater (1.97 raters per participant on average). Forty-seven participants recruited one rater; 184 recruited two raters; 20 recruited three raters; and 7 recruited 4–6 raters. In sum, we obtained 508 other reports.

### 2.2. Measures

#### 2.2.1. Self-Reports of Achievement and Power Motive

We used six items from the achievement and power subscales of the Unified Motive Scale (Schönbrodt & Gerstenberg, 2012). For achievement, participants rated the importance of different goals using a scale from 1 (not important) to 6 (extremely important). Example items are as follows: “Personally producing work of high quality” or “Projects that challenge me to the limits of my ability”. For power, three items were written as goals, which participants rated on a six-point scale, for example, “Be able to exert influence”. The remaining three items were statements such as “I would like to be an executive with power over others,” rated on a scale from 1 (completely disagree) to 6 (completely agree). Cronbach’s α coefficients were 0.88 (achievement) and 0.84 (power).

#### 2.2.2. Other Reports of Leadership Potential

We designed three items by drawing on the work on organizational reputation by Zinko et al. (2007): “This person has what it takes to become a successful manager in his/her future career”; “This person will be successful leading other people at a managerial position”; “This person will aspire to take on responsible duties in their future career”. We used all available ratings, even for those targets who had only one person rate their leadership potential. For targets with more than one rater, we calculated the mean ratings across raters for each target and the intraclass coefficients (ICC2). The intraclass correlation coefficient (ICC2) for other-rated leadership potential was 0.31, and Cronbach’s α of mean ratings was 0.80.

#### 2.2.3. Other Reports of the Overall Reputation

We used two items from Hochwarter et al. (2007) Reputation Scale: “This individual is regarded highly by others”; “This individual has the trust of his/her colleagues”. We used all available ratings, even for targets with only one person rating their overall reputation. For targets with more than one rater, we calculated the mean ratings across raters for each target and the intraclass coefficients (ICC2). The intraclass correlation coefficient (ICC2) for other-rated leadership potential was 0.39, and the Spearman–Brown coefficient of reliability was 0.53.

**Figure 1:**
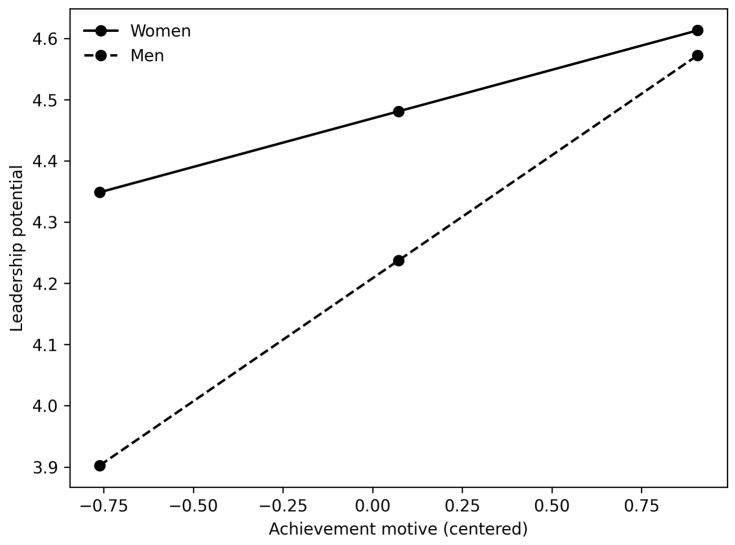
Interaction between achievement motive and gender in predicting leadership potential.

**Figure 2:**
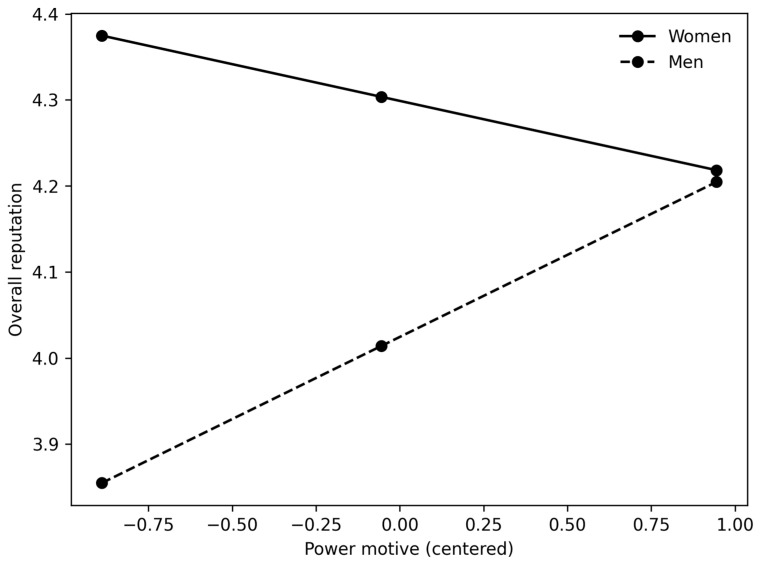
The interaction between power motive and gender in predicting overall reputation.

**Figure 3:**
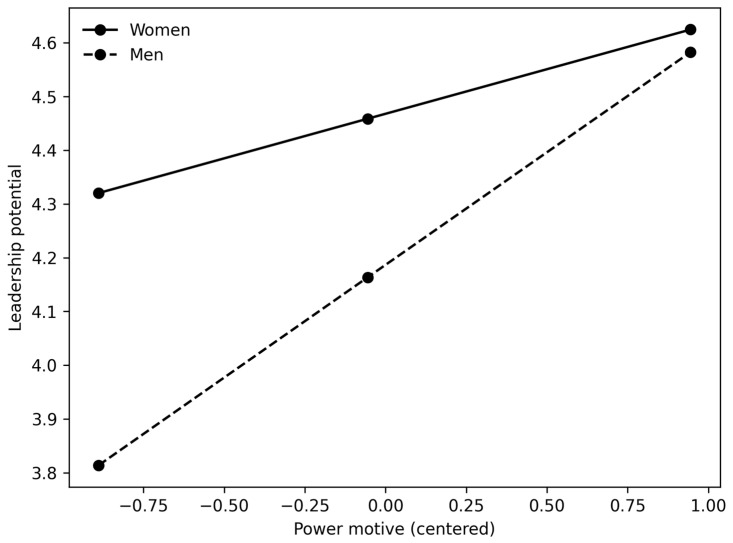
Interaction between power motive and gender in predicting leadership potential.

Generative artificial intelligence (GenAI) was used in this paper to generate Figures 1–3 in the Section 3. These figures were based on interaction data and code obtained from the PROCESS macro (Hayes, 2018) in SPSS 24 by IBM, New York, NY, USA. Additionally, Grammarly AI was employed to improve writing clarity and language precision.

## 3. Results

Table 1 presents the means, standard deviations, reliability coefficients, and correlations among all study variables. First, we can see that both achievement and power motive scores were around the midpoint of the scale. At the same time, other ratings of leadership potential and reputation were relatively high, suggesting that participants were generally perceived as competent and as having leadership potential. Second, achievement and power motives were moderately correlated (*r* = 0.60, *p* < 0.01), consistent with the idea that both reflect a basic motivation to get ahead. Similarly, reputation and leadership potential were moderately interrelated (*r* = 0.51, *p* < 0.01), suggesting that they are distinct yet related constructs. Third, females had slightly higher ratings of both overall reputation and leadership potential (*r* = 0.17, *p* < 0.01).

Finally, achievement motive was positively associated with other-rated reputation and leadership potential (*r* = 0.18, *p* < 0.05 and 0.27, *p* < 0.01). In contrast, power motive was significantly correlated only with peer-ratings of leadership potential (*r* = 0.32, *p* < 0.01). These results are in line with H1 when it comes to the relationships between achievement motive and other reports but only give partial support to the expected relationships between the power motive and other reports.

To examine the individual, unique predictive effects of achievement and power motives on other ratings of reputation and leadership potential (H2), we conducted two regression analyses (Table 2 and Table 3). In addition, to directly compare the strength of the predictors, we conducted *Z*-tests for differences in standardized regression coefficients and calculated the relative weight of each motive.

First, when it comes to overall reputation, the model accounted for a significant but modest (4%) percentage of variance, with achievement motive being the only statistically significant predictor and accounting for 90% of the explained variance. Even though we expected both motives to be related to overall reputation, the finding that the achievement motive is more important than the power motive is in line with our H2. Second, both motives were significant predictors of leadership potential, with a combined effect size of 11%. However, even though power motive had a higher relative weight (61% of the explained variance), we did not find a statistically significant difference in the strength of their contributions (z = −0.76). In sum, only achievement motive predicted reputation, and both motives were equally strong predictors of leadership potential. Therefore, we found partial support for H2.

Finally, to test whether the relationships between motives and other-reported reputational outcomes were moderated by gender (H3), we conducted moderation analyses. Before testing the hypothesized moderation effects, we also estimated a single comprehensive regression model that simultaneously included gender, achievement motive, power motive, and their interactions with gender, to assess the hypothesized effects while controlling for shared variance among the predictors. Although this model largely replicated the overall pattern of results, it exhibited substantial multicollinearity among predictors and interaction terms. Therefore, we proceeded with separate moderation models for each hypothesized relationship.

These moderation analyses were conducted using the PROCESS macro in IBM SPSS 24 (Hayes, 2018). We centered the continuous predictors (both motives) prior to the analyses. We centered the continuous predictors (both motives) prior to the analysis. In line with the correlational analysis, women had higher scores on overall reputation and leadership potential. Results in Table 4, Table 5, Table 6 and Table 7 show a trend in which the relationship between achievement and power motives and outcomes is slightly stronger for males in our sample. However, regarding the achievement motive, gender emerged as a significant moderator only in predicting leadership potential (*b* = −0.24, *p* < 0.05), but not overall reputation. Conversely, the interaction between gender and power motive was significant for both criteria (*b* = −0.28 and *b* = −0.25, *p* < 0.01).

Figure 1, Figure 2 and Figure 3 clearly show the difference in slopes. In both cases, even though women have a higher starting point (as shown by positive and significant regression coefficients between gender and leadership potential in Table 4, Table 5, Table 6 and Table 7), the regression slope is steeper for men, and in the case of the power motive and overall reputation, the slope for women even trends in the negative direction. These results are primarily consistent with our H3 and suggest that male participants high in power motive are perceived as having higher overall reputations and leadership potential. In addition, male participants who are high in achievement motive are perceived as having greater leadership potential. However, it should also be noted that the magnitude of the interaction effects is small, accounting for only 1.3–2.8% of the additional variance.

## 4. Discussion

Socioanalytic theory looks at personality from the perspective of the actor (person’s identity) and from the perspective of the observer (person’s reputation). Complementary to this, signaling theory suggests that people’s behaviors act as signals that convey their traits and intentions to others. This study builds on this theoretical framework. We focused on achievement and power motives, which might lie at the core of the need to get ahead in the socioanalytic theory, but have been rarely studied in this respect. These motives drive individuals to pursue challenging goals and influence behaviors that are likely to be noticed and interpreted by others and could lead to career opportunities (Zinko et al., 2012). Our goal was to test how these two self-reported motives (i.e., identity) shape a person’s reputation, operationalized through other-reported evaluations of (a) leadership potential—how ready they seem to obtain leadership positions in the future; (b) overall reputation—reflecting their general impressions of competence and trustworthiness. In addition, based on the role congruity theory (Eagly & Karau, 2002), we tested whether the relationships between the motives and reputational outcomes would differ between men and women.

We hypothesized that achievement and power motives would be positively associated with both reputational outcomes (H1). The results provided partial support for this hypothesis. Specifically, achievement motive showed significant positive correlations with both outcomes, whereas power motive was significantly associated only with leadership potential. Next, given the conceptual differences between the two motives, we further hypothesized that the achievement motive would be a stronger predictor of overall reputation and that the power motive would be a stronger predictor of leadership potential (H2). The regression analyses again provided partial support for the second hypothesis. Achievement motive emerged as the sole significant predictor of overall reputation, and both motives were equally strong independent predictors of leadership potential.

These results offer two takeaways. First, both motives emerged as equally strong predictors of other ratings of leadership potential among management students. It seems that both achievement and power motives lead to behaviors that can be interpreted as leadership-relevant: achievement-oriented individuals often demonstrate task proficiency and reliability. In contrast, power-motivated individuals exhibit assertiveness and influence attempts. These patterns correspond to the two broad behavioral antecedents of reputation: human capital (i.e., demonstrated skill/expertise) and social effectiveness (i.e., ability to influence and signal value) (Zinko et al., 2007). Although theory and research suggest that power motive has high importance in leader emergence and effectiveness (e.g., McClelland & Boyatzis, 1982; Judge et al., 2002; Galić et al., 2021), it seems that leadership potential is likely informed by reputational impressions built on both fronts—task execution and influence behavior—explaining why both motives are equally strong predictors. This could be especially true among university students. In this group, individuals may not yet have had the opportunity to lead formally, so that observers may rely on signals of diligence or ambition (achievement) and initiative or assertiveness (power) as equally valid indicators of leadership potential.

Second, achievement motive was the sole predictor of other ratings of overall reputation. The most likely reason for this is that achievement motive maps more directly onto the human capital dimension of reputation—associated with consistent, high-level performance, diligence, and goal orientation—behaviors that lead others to form positive, general impressions (Zinko et al., 2012). In contrast, the power motive, while potentially effective in emerging as a leader, does not inherently contribute to a broadly positive perception unless it is expressed through prosocial, socially skilled behaviors. Power-driven behaviors may sometimes deviate from normative expectations (e.g., being overly assertive or self-promoting), which can raise concerns about self-interest and reduce reputational favorability unless carefully managed (Ferris et al., 2007). Our results somewhat support this possibility—even though the effect was small and non-significant, the relationship between the power motive and overall reputation was negative after accounting for the shared variance between power and achievement. As Zinko et al. (2007) argue, reputations develop through positive deviation from norms. It may be that achievement-related behaviors are more consistently interpreted as such, especially in student settings.

Finally, we hypothesized that the positive relationships between the motives and reputational outcomes would be stronger for men than for women (H3). Our results show that (a) women had higher ratings of leadership potential and overall reputation (positive main effect of gender); (b) positive relationships between both motives and leadership potential were stronger for male participants; (c) regarding overall reputation, we only observed a significant interaction effect between gender and the power motive—again, the relationship was stronger for male participants. There was no interaction between gender and the achievement motive; (d) the moderation effect that we found was relatively small (explaining 1–3% of additional variance).

Drawing on socioanalytic theory (Hogan & Holland 2003), one possible explanation for better overall reputation and leadership potential for females is that women tend to score higher on communion-related traits, such as agreeableness, empathy, and interpersonal sensitivity (Costa et al., 2001). These traits are conducive to building positive peer reputations, as they signal warmth, reliability, prosocial behavior, and interpersonal competency, which may earn female students a more favorable reputation.

Furthermore, empirical evidence suggests that women are more lenient and generous in their evaluations of others than men (LaNoue & Curtis, 1985). It is possible that our sample included a high proportion of female students as both ratees and raters; it is plausible that gender-based leniency contributed to the observed difference.

However, male students gained more from exhibiting achievement and power motives, reinforcing a subtle form of gender bias, in line with the role congruity theory. Furthermore, one reason for observing the moderating effect of gender on leadership potential could be that evaluating leadership potential invokes gendered prototypes (e.g., the stereotypical leader as male), making observers more receptive to agentic signals from men and more skeptical of the same signals from women.

In contrast, overall reputation (general impressions of competence and trustworthiness) may involve less stereotypically masculine criteria, so women’s and men’s reputations benefited more equally from their traits, at least when it comes to achievement motivation. Interestingly, this was not the case for the power motive, where we found that male participants had a stronger positive relationship with overall reputation. One possible explanation is that achievement motivation signals competence without violating gender norms too intensively. In contrast, power motivation more directly signals dominance, which may be evaluated differently for men and women—at least when it comes to overall reputation for competence and trustworthiness.

### 4.1. Practical Implications

Our findings offer insights for HR practitioners. Both achievement and power motives carry important signals for building a reputation as “leadership material”. Individuals high in achievement motive typically signal their potential through consistent task performance and expertise. In contrast, individuals high in power motive signal their leadership aspirations by seeking influence—they may volunteer to lead projects, share ideas, or engage in networking. HR professionals and team leaders should be able to observe these signals in both the selection process (for instance, through situational tests and interviews) and talent development programs, and then use them to benefit the organization.

Understanding an individual’s motivational profile can guide personalized development plans. For those with a high achievement motive, development might focus on encouraging them to take on roles that increase their visibility and influence, so that key stakeholders notice their contributions. These individuals might benefit from mentorship in strategic networking or communicating their successes, ensuring their achievements translate into an enhanced professional reputation. On the other hand, our results suggest that high power motive does not necessarily translate into a favorable reputation of competence and trustworthiness. Therefore, individuals with a high power motive might naturally seek leadership roles, but they could benefit from coaching on how to pursue power in constructive, socialized ways.

Importantly, HR professionals, team leaders, and performance evaluators should generally be trained to interpret these contributions in a gender-fair manner. Structured assessment tools (such as leadership potential assessments or 360-degree feedback) can help ensure that evaluations of promotability consider objective accomplishments and demonstrated skills, thereby reducing the likelihood of bias from gender stereotypes. For example, a woman who is forthright about her ideas and career aspirations should be seen as equally indicative of leadership potential as a man exhibiting the same behavior—and HR can play a role in communicating this principle to supervisors and performance-evaluators.

### 4.2. Limitations and Directions for Future Research

Even though our results provide valuable insights for both research and practice, this study has several limitations. First, our sample consisted of management students in a university setting, which limits the generalizability of the findings to “real” workplaces. Professional workplaces often have more diverse age groups, more pronounced power dynamics, and higher stakes associated with reputation. Consequently, the formation and impact of reputation in our study might differ in strength from that in real organizations. Given these considerations, this study should be viewed as a proof-of-concept study in which a student sample is appropriate for testing initial hypotheses, even though it limits the generalizability of the findings. Future research should test whether our findings can be replicated in samples of working professionals across different industries, thereby establishing their ecological validity.

Second, we conceptualized reputation into two broad categories, used short measures (two and three items per category), and obtained low inter-rater reliabilities and relatively low internal consistency (SB = 0.53) for the two-item scale assessing overall reputation. Limited reliability likely attenuated the observed relationships between predictors and reputation, and it may also indicate that the scale has limited construct validity, as it likely does not capture the full breadth of the underlying construct of reputation. Related, the observed significant interaction terms, though statistically significant, were small in size and should be interpreted with caution and replicated in future studies.

Future research could also focus more on specific domains of reputation, such as task competence, sociability, or integrity, while utilizing longer, more detailed instruments. In addition, it is possible that the raters (participants’ knowledgeable others) gave them favorable ratings, as the means were relatively high (4.22 and 4.38 on a 5-point scale). This ceiling effect could have reduced variability and, therefore, the obtained effect sizes.

Third, the present research focused only on the need to get ahead, but socioanalytic theory also emphasizes the importance of getting along. Therefore, there is also an opportunity to study the affiliation motive alongside the power and achievement motives. It could be exciting to see whether men and women differ in relationships involving this motive and in different facets of reputation. Finally, it would be helpful to study the formation of reputation in longitudinal research, as well as studying how reputation translates into work and career outcomes.

## Figures and Tables

**Table 1:** Descriptive statistics and correlations between key variables.

	*M (SD)*	1.	2.	3.	4.	5.
1. Gender (Male = 1, Female = 2)	-	-				
2. Achievement motive	3.69 (0.85)	0.09	(0.88)			
3. Power motive	3.16 (0.92)	0.04	0.60 **	(0.84)		
4. Overall reputation	4.22 (0.73)	0.17 **	0.18 *	0.02	(0.53)	
5. Leadership potential	4.38 (0.75)	0.17 **	0.27 **	0.32 **	0.51 **	(0.80)

Note: Internal consistency coefficients are on the diagonal; * *p* < 0.05, ** *p* < 0.01.

**Table 2:** Regression analyses of the achievement and power motives in predicting overall reputation.

	Overall Reputation					
Predictors:	*b (SE)*	*β*	*RW*	Lower 95% *CI*	Upper 95% *CI*	*p*
Achievement motive	0.23 ** (0.07)	0.26 **	0.90	**0.10**	**0.36**	0.00
Power motive	−0.11 (0.06)	−0.13	0.10	−0.23	0.02	0.09
*R* ^2^	0.04 **					
*Z*-test	3.7 **					

Note. ** *p* < 0.01. *RW* = relative weights, *CI* = confidence intervals, *CIs* that do not contain zero are in boldface.

**Table 3:** Regression analyses of the achievement and power motives in predicting leadership potential.

	Leadership Potential					
Predictors:	*b (SE)*	*β*	*RW*	Lower 95% *CI*	Upper 95% *CI*	*p*
Achievement motive	0.13 (0.07)	0.14	0.39	−0.06	0.26	0.06
Power motive	0.20 ** (0.06)	0.24 **	0.61	**0.08**	**0.31**	0.00
*R* ^2^	0.11 **					
*Z*-test	−0.76					

Note. ** *p* < 0.01. *RW* = relative weights, *CI* = confidence intervals, *CIs* that do not contain zero are in boldface.

**Table 4:** Moderated regression analysis—gender × achievement motive in predicting overall reputation.

	Overall Reputation				
Predictors:	*b (SE)*	*β*	Lower 95% *CI*	Upper 95% *CI*	*p*
Achievement motive	0.51 ** (0.19)	0.57 *	**0.13**	**0.89**	0.01
Gender (Male = 1, Female = 2)	0.26 ** (0.109)	0.17 **	**0.07**	**0.44**	0.01
Achievement motive × Gender	−0.21 (0.11)	−0.41	−0.44	0.08	0.06
*R* ^2^	0.07 **				
Δ*R*^2^	0.013				

Note. * *p* < 0.05, ** *p* < 0.01, *CI* = confidence intervals, *CIs* that do not contain zero are in boldface.

**Table 5:** Moderated regression analysis—gender × achievement motive in predicting leadership potential.

	Leadership Potential				
Predictors:	*b (SE)*	*β*	Lower 95% *CI*	Upper 95% *CI*	*p*
Achievement motive	0.64 ** (0.19)	0.71 **	**0.26**	**1.03**	0.00
Gender (Male = 1, Female = 2)	0.26 * (0.10)	0.17 *	**0.07**	**0.45**	0.01
Achievement motive × Gender	−0.24 * (0.11)	−0.46 *	**−0.47**	**−0.02**	0.03
*R* ^2^	0.12 **				
Δ*R*^2^	0.016 *				

Note. * *p* < 0.05, ** *p* < 0.01, *CI* = confidence intervals, *CIs* that do not contain zero are in boldface.

**Table 6:** Moderated regression analysis—gender × power motive in predicting overall reputation.

	Overall Reputation				
Predictors:	*b (SE)*	*β*	Lower 95% *CI*	Upper 95% *CI*	*p*
Power motive	0.47 ** (0.17)	0.58 **	**0.13**	**0.80**	0.01
Gender *(Male = 1*, *Female = 2)*	0.27 ** (0.10)	0.19 **	**0.09**	**0.46**	0.00
Power motive × Gender	−0.28 ** (0.10)	−0.58 **	**−0.48**	**−0.08**	0.01
*R* ^2^	0.06 **				
Δ*R*^2^	0.028 **				

Note. ** *p* < 0.01. *RW* = relative weights, *CI* = confidence intervals, *CIs* that do not contain zero are in boldface.

**Table 7:** Moderated regression analysis—gender × power motive in predicting leadership potential.

	Leadership Potential				
Predictors:	*b (SE)*	*β*	Lower 95% *CI*	Upper 95% *CI*	*p*
Power motive	0.67 ** (0.17)	0.81 **	**0.34**	**1.00**	0.00
Gender (Male = 1, Female = 2)	0.28 * (0.09)	0.18 **	**0.10**	**0.47**	0.00
Power motive × Gender	−0.25 * (0.10)	−0.51 **	**−0.44**	**−0.06**	0.01
*R* ^2^	0.15 **				
Δ*R*^2^	0.022 *				

Note. * *p* < 0.05, ** *p* < 0.01, *CI* = confidence intervals, *CIs* that do not contain zero are in boldface.

## Data Availability

The data presented in the study are openly available in Zenodo at https://doi.org/10.5281/zenodo.17588650.
